# The Influence of Physical Agents on Life

**Published:** 1833-01-01

**Authors:** 


### Medico-Chirurgical Review,

No. XXXV.

OCTOBER 1, 1832, TO JANUARY 1, 1833.

### I.

On the Influence of Physical Agents on Life. By Dr. *Edwards*. Translated from the French, with Notes by Dr. *Hodgkin*, and Dr. *Fisher*. 8vo. pp. 488. Highley, London.

THE object of Dr. Edwards' researches is to examine and ascertain the effects produced by air, water, temperature, light, and electricity, those agents, whose operation is continually, although often imperceptibly, exerted on the life of different animals. It is true that they almost always act simultaneously and conjointly; and hence it is no easy affair to discriminate the influences of one alone from those of the others; the air, besides its elementary constituents, is charged with moisture and electrical fluid; the quantity of heat and of light too is ever varying, so that in our inquiries into the agency of the atmosphere on animal existence, we ought to consider it under the conditions, not only of its chemical purity, of its quantity, motion, rest, weight, and rarity, but also of its dryness and humidity, of its strong and feeble electricity, of its translucency and darkness, and of its degrees of temperature.

Dr. Edwards has laboured most meritoriously and with the most gratifying success in elucidating the influence exerted by each of these physical powers, separately and conjointly, and the present work is the record of the almost countless experiments which he has performed, and of the conclusions to which these experiments have conducted him. It is in truth a rich granary of curious and interesting facts, collected for a series of years with unwearied industry: and as most of his statements have been confirmed by the researches of others, we may place the most implicit reliance on their authenticity and correctness. So high is our opinion of the author's talent and ingenuity, that we regard him quite as a pattern and model to all physiological experimenters; his are not the once or twice observed phenomena, and the rapid inferences; the single fact, or fact supposed, and the widely spreading hypothesis, but the oft-repeated, and varied trial, on numerous animals of different classes and of different modes of existence, and the cautious and slow inductions of a truly philosophical mind. He began his researches, by ascertaining the influence of the physical agents on the lower orders of vertebrated animals, and proceeded up the scale to man. We shall follow him as closely as possible, in order that our readers may have a faithful summary of his labours.

It has been long known that animals of inferior organization are, generally speaking, less affected, and less dependent on external influences than those of a higher grade; that they have greater tenacity of existence; that this existence has more and more of a vegetable character, stronger in itself, as a whole, and better able to withstand rude shocks and injuries; and also, stronger in the separate vitality of its individual parts, so that they will often continue to live even when they are dismembered from the parent stock.

Dr. Marshall Hall has very recently published some interesting observations on the mutual connexion, or at least, co-existence of high or low irritability among animals, with a state of less, or mere perfect development of the function of respiration. By the term "irritability" he means the power of retaining vitality, under the privation of food and air, and after the infliction of any mutilations. We shall allow him to speak for himself.

"The quantity of respiration is inversely as the degree of irritability, and the facts, that the activity of the animal is directly as the former, and that its tenacity of life under the privation of air, food, and other stimuli, is directly as the latter. The very young animal has a lower respiration, and a higher irritability than the adult. It has less power of evolving heat, and greater power of bearing the privation of air.

The adult animal has a higher respiration, and a lower irritability. It has greater activity, and less tenacity of life, under the privation of air and food.

The animal which maintains a steady given temperature, has a higher respiration, and a lower irritability, in winter than in summer. Animals which do not maintain their temperature, have a lower respiration, and a higher irritability, in winter and summer. When the cold induces the state of torpor, these phænomena are observed in a still more marked degree, and the animal bears asphyxia, and the privation of food, with comparative impunity." 469.

We have already stated, that Dr. Edwards commenced his researches on the lower classes of animals, and gradually ascended the scale of organization; his first enquiries are, on the influence of the atmosphere on the batrachian reptiles, such as frogs, toads, and salamanders.

"I strangled six frogs, by tying, very tightly, with a packthread round the neck, a piece of bladder fitted very closely to the head, so as to exclude the air. In fact, the ligature was sufficiently tight to effect this of itself. At first the frogs were paralysed, but they afterwards to a great degree, recovered, and lived from one to five days." 11.

He repeated the same experiment on salamanders, with similar results.

"One of these animals lived twelve days, when the head became gangrenous; it afforded me an opportunity of making observations analogous to those of M. Dumeril, in his interesting experiments on a salamander, which survived decapitation a sufficient length of time for the neck to cicatrize." 12.

If instead of strangulation, we drown a frog in distilled water, life is extinct in a very much shorter space of time; hence we may infer that the air exerts a vivifying influence on the nervous system, and probably also on the cutaneous circulation of the animal. Dr. E. suspected that the water might be positively injurious per se, as well as by excluding the breathing; and in order to ascertain this, he annihilated the functions of circulation and of respiration, by cutting out the hearts of salamanders, and afterwards exposing some of the animals to the air, and immersing others in distilled water of the same temperature.

"In about four or five hours the salamanders in the water appeared dead; but that life still existed was rendered evident, when they were moved or pinched. One died in eight hours, the other in nine. Those in the air, however, lived from twenty-four to twenty-six hours." 10.

Again:—

"If a frog, thus deprived of its heart, and immersed in water, be drawn out and exposed to the air, at the moment when all signs of life have disappeared, it immediately begins to recover. If it be again plunged in water, all appearance of life instantly ceases; and it may thus be made, several times alternately, to lose and recover its motion and sensibility." 10.

We may, therefore, infer that air, in comparison of water, has a superior vivifying influence, or at least acts less deleteriously on the nervous and muscular system of batrachian reptiles, independently of its action on the functions of respiration and circulation. The application of this discovery to any reasonings on the asphyxia of these animals, induced by strangulation or by drowning, is too obvious to be commented upon; and we are thus led to appreciate the importance of a comprehensive and accurately ascertained knowledge of facts, and of most cautious speculation, in all physiological enquiries.

Our readers will, no doubt, perceive that even the experiments of Dr. Edwards, hitherto enumerated, do not quite satisfactorily exhibit the simple and unmodified effect of interrupted breathing on the batrachian animals, since a mechanical injury was inflicted, at the same time, by tying a ligature round the neck. In order to obviate this objection, and to ascertain simultaneously the influence of the air on the cutaneous circulation, he inclosed the frogs, toads, and salamanders in a solid material, which should exclude the air from the lungs, and also from the skin, and which should exert no deleterious influence on the nervous system. He filled five small boxes with plaster, and imbedded a toad in each of them; they were all alive on the 19th day afterwards. Frogs, placed in dry sand, were also found perfectly alive after many days.

Now, what at first view appears strange is, that if these animals be exposed to the air in small dry bottles, they will be observed to die much more speedily than those which had been encased in the plaster, or sand. The explanation of this occurrence is proved by Dr. E. to be—that, in the former case, they rapidly waste, and undergo complete desiccation. The more rapid death is, therefore, to be attributed to the greater loss of the fluids by perspiration, the truth of which is readily proved—

"By exposing some frogs to the air in dry vessels, and burying others in dry sand, and afterwards weighing them, at intervals of two, three, four, and five days, I uniformly observed a greater loss in the air than in the sand. Comparative experiments were also made in air and plaster upon toads, and the difference was much more striking than in the sand. Hence the cause of the greater duration of life in sand or plaster than in air, is from the perspiration being more abundant in the air than in the solid substances.

Under an exhausted receiver, in which the effects of rapid evaporation and absence of air are combined, death, as might be expected, takes place very speedily." 15.

Before we quit this part of the subject, it is of importance to mention, that Dr. Edwards has most satisfactorily ascertained that carbonic acid is secreted by the skin in frogs; thus a most intimate association and sympathy of function between the lungs and external surface exist, at least in these animals.

Several of the above observations are probably new, and all of them, no doubt, interesting to our readers; but the following are still more curious, and, we trust, will arrest their studious attention, as they are admirably fitted to throw light on one of the innumerable, wonder-working operations of animate nature.

In the months of July and September, when the temperature of the atmosphere was from 60° to 58°, several frogs were drowned in Seine water, the temperature of which was 60°; the mean duration of life, or of sensibility to stimuli, was 1 hour and 37 minutes in July, and 1 hour, 45 minutes, in September. But if the water was cooled down to 50°, the frogs were found to live from 5 to 6 hours; if to 32°, from 7 to 8 hours, 18 minutes. If, on the other hand, the temperature was raised to 72°, the frogs lived only from 70 to 35 minutes; if to 90°, from 32 to 12 minutes; and if to 108°, they lived scarcely a few seconds, and in no instance for more than 2 minutes.

It appears, therefore, that the temperature most favorable to the continuance of the life of frogs, immersed in water, is that near the freezing point; but we shall find that, in conducting such experiments, we must pay regard not only to the agency of the means we employ, but also to the season of the year at which we perform them. We have already seen that, when frogs were drowned in water of 60°, during July and September, the average continuance of their lives was about 1 hour and 40 minutes; if the same experiment be made in the month of November, Dr. Edwards found that the frogs lived from 2 to 5 hours and a half. All the circumstances being the same in these cases, except the season, it is to this cause that the difference in the results must be ascribed; and, in order to be more completely satisfied of the truth of this, we have only to continue our observations later in the Winter, and we shall find a uniform and progressive manifestation of the same phenomena. Thus,

"On the 22d Dec. the thermometer having been about 0° cent. or 32° Fahr. for twenty days, three frogs were put in water at 10° cent. or 50° Fahr.; they lived from twenty to twenty-four hours. On the 23d Dec. the temperature being still 0° cent. or 32° Fahr. four frogs were placed in water at 0° per cent. or 32° Fahr. the same apparatus being employed as in the preceding experiments. They lived from twenty-four to sixty hours." 20.

We are forced, then, to the conclusion, that the system or constitution of the batrachia undergoes a considerable change, during the year, in the amount of its irritability or vital powers; the change, in all probability, is a gradual one during the warmth of Summer, coolness of Autumn, and the cold of Winter, at which season these animals are found to be most tenacious of existence, and, therefore, capable of resisting for a longer time all influences which are destructive of it. Perhaps there is more air contained in the water during the Winter season—but this is conjectural, and we must confine ourselves strictly to what is known to be true. That the air contained in water, at ordinary temperatures, has a very marked influence on the duration of the life of frogs, when immersed in it, is readily proved; and it appears, from Dr. Edwards' experiments, that when frogs can live from six to ten hours in a certain quantity of aërated water, they die in from three to five hours in water that has been boiled. But the length of time that a frog will continue to live in aërated water depends, in a very remarkable degree, on whether the water be renewed at intervals or not. Dr. E. in the month of December, immersed a frog in a glass vessel, which held 17 pints of Arcueil water; it was drawn off by a syphon every day, only leaving sufficient to cover the animal, and was replaced by fresh. This frog continued to live for more than two months and a half, and its death appeared to be occasioned by an accidental neglect to renew the water.

"This experiment shews the remarkable fact, that frogs are really amphibious, since they can not only breathe the air of the atmosphere, but can also live exclusively by means of the air contained in water.

Tadpoles, which are possessed of gills as well as lungs, can also live in water without coming to the surface; a fact which I proved by an experiment, conducted in the same manner as that which has just been detailed. They cannot, however live on land previous to the full development of their limbs." 27.

It may be enquired, on what part does the water, or rather the air in the water, act: is it admitted into the lungs, or is its operation limited solely to the skin? That the former is not the case is proved, by all observers having remarked that, when a frog is plunged into water, it is extremely rare that any movement of deglutition (our readers probably know that frogs swallow, and do not respire air) can be seen; and Dr. E. to establish this still more conclusively, has most carefully examined the lungs after such experiments, and in no instance has he ever detected any water in them. We must, therefore, refer the action of the aëration to the skin, the only other organ in contact with the fluid. That the blood is actually aërated is shewn by the fact, that the contents of the arteries, in the webs of the feet, are evidently florid.

From the foregoing details we are authorized in concluding, that the duration of the life of batrachian animals, when immersed under water, varies exceedingly—first, according to the presence or want of air in the water; secondly, on the quantity, or, what is equivalent, the renewal of the water; and, lastly, according to its temperature, and to the season of the year at which the experiments are performed. We have seen that, in a small quantity of water, even when aërated, they live only for a few hours; that if the water be changed every day, the life may be prolonged, provided always the temperature is not much elevated. So striking is the influence of this last agency, that if we immerse frogs in two gallons of water, of a temperature between 32° and 50°, they may be preserved a long time alive; but if the heat be raised only two or three degrees, the animals will much more speedily die, unless the quantity of air be increased, by furnishing, in a given time, a greater quantity of aërated water; and the correctness of this statement is beautifully confirmed, by finding that frogs, immersed in running water, can resist an elevation of temperature, which would be fatal to them in vessels if the water be changed only once a day. Although these animals can live under water of a low temperature, we must not suppose that they retain all their natural alacrity of existence.

"The extreme activity of frogs is well known, and there is a striking contrast in this respect between them and toads; but keeping them under aërated water destroys this characteristic. It does even more; they become so sluggish in their movements as to resemble tortoises. The slightest noise, which in their state of liberty excites a panic among them, at that time makes no impression. Light, which, on other occasions calls them so easily to the surface, no longer induces them to rise, when the temperature is sufficiently low. They have, however, the faculties of sense and motion; but in air of the same temperature they are extremely lively." 34.

We regret that Dr. Edwards has not carried his experiments so far as to ascertain the absolute longest period that frogs can live under water in the most favourable circumstances; we cannot believe that they can ever be made altogether independent of pulmonary aëration, especially, as we find that a residence of six or eight weeks induces such a sluggishness of vitality.

In order to determine the comparative importance of the pulmonary and cutaneous functions, Dr. Edwards instituted the following experiments. On one occasion he gagged six frogs, the temperature (and let this be observed) being 75°. Five died on the following day, and the remaining one lived till the 7th. At another time he strangled the same number of frogs by applying a ligature tightly round the neck, the thermometer standing at 53°; they were placed on wet sand. They lived a considerable time [the exact duration of time ought certainly to be stated—ED.] one of them for twenty days. En passant, we suspect that Dr. Hodgkin has not very faithfully translated the original of Dr. Edwards, or at least, has omitted the details of some experiments, whereby confusion has been introduced; for example, at page 11, it was said, as our readers will observe in the extract we have given them, that frogs, when strangled, lived only from one to five days, whereas, we are now told, at page 36, that they have survived for twenty days. There appears much incongruity in these two statements, and they can be reconciled only by supposing that, in some other respects, perhaps that of temperature, one of the most influential agents, the experiments were not identical.

Not satisfied with either of the above methods, Dr. Edwards cut the lungs completely out, which may be done by a very slight incision, and with little loss of blood.

"I performed this extirpation in the middle of December, 1818, on three frogs of moderate size. They did not appear to suffer much, and presented, after the operation, the same activity as those which had not been touched. I placed them upon moist sand. The temperature of the room was 7° cent. or 45° Fahr., and it rose to 12° cent. or 53° 6′ Fahr. on the 17th Jan. 1819. Two died at this time, having lived thirty-three days, and the third on the 24th, having lived forty." 37.

It appears, therefore, that the skin of itself is insufficient to purify the blood. Let us now see whether the lungs can effect this without the co-operation of the cutaneous function. A frog was placed in a glass, holding five ozs. of water; a lid, with a central opening, was laid on the surface of the water, so that the only point at which the air came in contact with the animal, was at the opening, where it breathed. The water was changed every day, and it lived for three months and a half. It may be objected that the air contained in the water acted on the skin. Another method was therefore employed. The skin was coated with different varnishes, but these did not adhere: oil would exclude the air, if it were free from objection in other respects. If it be substituted for water in the glasses with perforated lid, the frogs were found to die in from 7 to 20 hours. We are surprized to be told in the next sentence, that if some frogs be drowned in vessels holding $5\frac{1}{2}$ ounces of oil, and others in the same quantity of water, and not allowed in either case to breathe, they lived equally long in both liquids! How does this observation tally with its next door neighbour?—"This substance, viz. oil, has a deleterious action on the skin." We advise Dr. Hodgkin to re-examine and compare these and other passages, in the event of a second edition being required. The conclusion is, in all probability, a just one, that the lungs alone in the batrachia, unassisted by the skin, is incompetent for the aëration of the blood, although we must acknowledge that the experiments on this head are not nearly so satisfactory, as those which have reference to other subjects. We have already illustrated the very striking influences of different temperatures, on the life of frogs, but as yet we have not attempted to explain, or account for these; perhaps one among several reasons, is, that the higher the temperature may be, the greater is the loss by perspiration, and this loss is not compensated by equivalent absorption. The skin of the batrachia seems to be an active exhaling and absorbing surface; the rapid loss of weight of a frog, exposed to dry air, abundantly proves the former, and its speedy recovery of the lost weight, when immersed in water, equally demonstrates the latter proposition; the preponderance of the one function over the other depends much on the temperature. It is stated by Dr. E., that—

"It appeared from experiments, that at 0° cent. or 32° Fahrenheit, the absorption predominates over the loss of weight; while at 30° cent. or 86° Fahr. the losses are greater than the increase by absorption. It was also observed that elevation of temperature in water had a marked tendency to augment the animal excretions; from which we seem authorized to conclude that an analogous effect would be produced upon perspiration in the air." 50.

We have now finished our analysis of that portion of Dr. Edwards' work, which treats of the influences of air and water on the life of the batrachian reptiles, and proceed to the second part, which is devoted to a similar examination of fishes, and the remaining orders of reptiles, viz. lizards, serpents and tortoises. But we shall first offer a few remarks on the effect of some physical agents on the transformation of tadpoles into perfect animals. Our readers are probably aware, that tadpoles unite, in regard to respiration, the functions of reptiles with those of fishes, from their possessing two kinds of breathing organs, viz. lungs and gills; hence they are more independent of the external air than frogs, and can continue to live under water in more unfavourable circumstances, as for example, in higher temperatures, without having recourse to the surface to breathe; and if we so conduct our experiments, that the tadpoles are kept constantly under water, we find that they undergo the change from a larva to a complete animal, very slowly, imperfectly, and probably often not at all. To demonstrate this—

"I procured a tin box, divided into twelve compartments, each of which was numbered and pierced with holes, so that the water might readily pass through the box. A tadpole, (which had been previously weighed) was put into each compartment, and the box was then placed in the river Seine, some feet below the surface. A larger number was at the same time put into an earthen vessel, containing about four gallons of Seine water, which was changed every day. These tadpoles were at liberty to rise to the surface and respire air, and they soon went through their metamorphosis. Of the twelve placed in the box under water, ten preserved their form, without any progress in their transformation, although some had doubled, and others trebled their weight. It should be observed, that at the time when the experiment was begun, the tadpoles had acquired the size at which the change is about to take place. Two only were transformed, and this very much later than those which, in the earthen vessel, had the liberty of respiration in air." 53.

In drawing any conclusions from this experiment, we must keep in mind, that the tadpoles were deprived of the genial influence of light, an agent of primary importance in the development of all organized forms. It is a fact of great interest, that the proteus, (which, our readers are perhaps aware, and the siren, are the only two strictly amphibious, that is, having lungs and gills, animals known) has been always found placed in those conditions, as to temperature, darkness, and respiration, which we have shewn above, are those in which the branchiæ are likely to remain ; it inhabits the subterranean waters of the lakes of Carniola. Before quitting this subject, we must not omit to state, that the quickness of the transformation of tadpoles is much influenced by the quantity of nourishment afforded to them; it may be greatly accelerated by feeding them with crumbs of bread and other nutritious substances, especially if the season be warm and favourable. As a general observation, it may be asserted that the more genial the temperature, the more nutritious the food, and the more exposed, at least within certain limits, they are to the external air, the change is proportionally more speedily effected, and vice versâ.

### CHAP. II.—ON THE LIFE OF FISHES, AS AFFECTED BY PHYSICAL AGENTS.

If fishes be kept in water deprived of air, they quickly die; but the period varies, as in the case of the batrachia, according to the temperature of the fluid; the lower the heat the longer they live. In water at 104°, they scarcely ever live more than two minutes. Even in aërated water, provided the quantity be small, exposed to the atmosphere, the duration of life must depend on the temperature; for example—

"If we put a bleak (cyprinus alburnus) into a vessel with a large mouth, containing five ounces and a half of aërated water at 20° cent. or 68° F. in summer, it dies within a few hours : but when the temperature is lowered to 10° or 12° cent., or 50° or 53° F., and is kept at that degree, the animal lives until its secretions are so abundant as to corrupt the water. If, to remedy this inconvenience, we merely renew the water every twenty-four hours, the animal lives in it almost indefinitely." 58.

Now this is exactly what we have above stated to take place with frogs; between 32° and 50°, they live an indefinite time in aërated water, if it be renewed sufficiently often ; but they speedily die, if the temperature exceeds this limit. Let us now see how they are affected, and to what extent their vitality is influenced by the atmosphere. Sylvestre ascertained that fishes died more quickly in vessels where the surface of the water was excluded from the air, than in those where the surface was exposed; we therefore infer that it is necessary for fishes that they should come occasionally to the surface to breathe. To discover the comparative importance of the bronchial and cutaneous functions, Dr. Edwards performed numerous experiments, which have certainly thrown some, but not much, light on the subject. If a fish be taken out of the water, the gills continue to move for a shorter or a longer time, the skin becomes more and more dry, and, at the time of death, it is frequently stiff, and, as it were, quite desiccated. Dr. Edwards found that the fish loses during this time, by perspiration, a fourteenth, or thereabouts, of its weight. We will readily concede to our author, that such a loss may certainly accelerate the death of the animal; but we suspect that few of our readers will agree with him in the belief, "that the loss which fishes experience by evaporation, is enough to cause their death in air;" and, indeed, the experiments which are next detailed at once negative such an idea.

"A fish, which had been wiped and then weighed, was suspended in a limited quantity of aërated water, so that it had its head and gills above the surface; it died in nine hours and twenty-one minutes. On then weighing it again, it appears that it had not sensibly diminished in weight, but on the contrary had slightly increased." 62.

And again:—

"I placed some fishes in the opposite position to that of the fish employed in the last experiment, that is, with the head and gills in water of the same quality and quantity, and the trunk, suspended in the air by a thread passed through the end of the tail. They lived in this state many days. I weighed them after that interval, and discovered that there was evidently, in this case, a slight increase of weight. But the drying of the surface of the part of the trunk exposed to the air was as marked as in the case where these animals were entirely exposed to the atmosphere, and where they died after a considerable diminution in weight. It is therefore evident that the fluid absorbed by the gills was not distributed to the rest of the body in a proportion sufficient to repair, in all parts of the trunk, the loss which it had sustained by perspiration in air." 63.

The statement, therefore, that fishes die from the quantity of water which they lose by perspiration, appears to us most strange, and to be quite contradicted by the author's own shewing. We are surprized that he did not think of the simple experiment, of continually moistening the whole surface of a fish taken out of water; for, according to his view of the subject, a fish so treated should have continued to live for many days, or even months, yet we need not say how different is the result. We regret that the original of Dr. Edwards' work is not before us; for it seems most improbable that such an acute observer, and so rigid an adherer to facts, can have so heedlessly arrived at conclusions, apparently at complete variance with his own experiments.

Chapter 3d treats of the remaining divisions of reptiles, viz. lizards, serpents, and tortoises; it appears to us to be exceedingly imperfect, and we suspect it has been injudiciously curtailed by the translators. The few facts worth notice we shall present to our readers. The lungs are sufficient for the aëration of the blood in the two latter classes; in lizards, on the contrary, the action of the air on their external surfaces is found to be necessary to life. Dr. Edwards accounts for this difference very ingeniously, by the less ample and capacious volume of the lungs in the saurian, than in either the ophidian or chelonian reptiles; he found that the larger the lungs were, the animal was so much more independent of cutaneous aëration. This latter function is of much less extensive influence than in the batrachia—it can no longer compensate for that of the lungs; and we accordingly find that neither lizards, serpents, or tortoises, can be made to live under running aërated water, even when accompanying circumstances are most favourable. The quantity of perspiration has also much diminished; the skin has become scaly or laminated; and from these differences, arises the variety which we observe in the duration of the life of these animals, when deprived of nourishment.

"This diversity (says Dr. Edwards) depends on the rapidity or slowness of perspiration, as is proved by the numerous and varied experiments which I have made on the duration of the life of batrachians, under different circumstances with respect to perspiration, among which the effect of solid coverings was the most remarkable. The influence of temperature on the duration of life in lizards, serpents, and tortoises, is analogous to that which I have already shown to be the case with batrachians and fishes." 67.

The second great division of vertebrate animals, viz. those whose temperature is steadily above that of the surrounding medium, and which, therefore, are denominated warm-blooded, comes now under our consideration, in order that we may ascertain to what extent, and in what manner, they are influenced by these physical agents, whose operation on cold-blooded animals, including all reptiles and fishes, has been already investigated. And, first, we shall endeavour to establish the most prominent and important phenomena, connected with the production of their animal heat. It has been very generally believed, that the temperature of young animals is higher than that of adults; but the belief has not been drawn from experiments, so much as from reasoning on the greater activity of their circulation and nutritive energies. Dr. Edwards has repeatedly examined the heat of young puppies, rabbits, kittens, and other animals, and has never found that it is higher, but generally one or two degrees, or even more, below the standard of the parent. The temperature of very young birds is also less than that of the hen; and the remark is equally true in reference to young children.

"In ten healthy infants, from a few hours to two days old, in the wards of an hospital, under the care of my friend M. Breschet, the limits of variation were from 34° to 35° cent. or 93° to 95° Fahr.; the mean of the whole number was 34° 75 cent. or 94° 55 Fahr. Their temperature is, therefore, inferior to that of adults; a relative difference rendered probable by analogy, and confirmed by observation." 115.

And, from the following examination, it appears that, in prematurely-born children, the heat of their bodies is less and less, in proportion as the period of their birth recedes from the full term of fœtal life.

"Through the kindness of Dr. Dagneau, who attended a lady who was my patient also, I had an opportunity of ascertaining the temperature of a healthy seven-months' child, within two or three hours after birth. It was well swathed, and near a good fire, but the temperature at the axilla did not exceed 32° cent. or 89° 6′ Fahr." 116.

Let us now ascertain, whether new and recently-born animals have the same power of retaining their heat as adults have. Dr. E. tells us, that—

"If, at the temperature between 10° and 20° cent. or 50° and 68° Fahr., a new-born puppy be removed and kept an hour or two from its mother, its temperature falls considerably, and continues falling until, in the course of three or four hours, it stops a very few degrees, above that of the surrounding air." 69.

And again :—

"Another series of experiments was made when the air was 22° cent. 71° 6′ Fahr. Even at this high temperature sparrows of the same age cooled rapidly to within one degree of the atmospheric temperature. It is true that these birds are hatched without feathers, but feathers are merely a covering; although they may *retain*, they cannot *produce* heat. It is from within alone that animal heat can originate, however outward coverings may contribute to retard its dissipation." 71.

The same is true of young hawks, which, though covered with a thick down, and almost as large as pigeons, in an atmosphere of 62°, suffered a diminution of 26° when taken from the nest; hence we infer, that some young birds have an inferior power of generating heat, and are, therefore, less capable of resisting cold. This position is undoubtedly true, but is not of universal application to the progeny of all warm-blooded animals ; for example, young guinea-pigs have a heat nearly as high as that of adults, and their temperature does not fall, even when they are withdrawn from the mother. Some birds (the individuals are not stated) also can, as soon as they are hatched, maintain an elevated temperature, even when exposed to the air; they come into the world in a more advanced state than other birds, for, soon after leaving the shell, they can eat and run about, and it is when other birds can do the same, that they also develop the same degree of heat. The young birds which are able to run about, and which retain their heat, are only covered with a tolerably thick down, and not with feathers. If we turn our attention to the young of the human race, we shall find that a new-born infant, born at the full time, has the power of maintaining nearly a uniformly high temperature when separated from the mother ; it becomes, therefore, a question of curious interest, to what cause are we to refer the different capacities of the young of birds and mammalia, in respect to preserving or losing their natural heat; and to enquire also, if there be any resemblances in organization which denote and distinguish the two divisions of animals. That the true cause is not to be found in the differences of their skin-coverings, is abundantly obvious; the young child is unprovided with such ; and although rabbits (which, as stated above, belong to the cold-blooded division) are born almost naked, puppies and kittens are well covered with hair, and even if we add an artificial covering, little perceptible change results. Dr. Edwards, after much reflection, perceived that a certain relation might be traced between the state of the eyes, at the time of birth, and the power which the young animal has of maintaining a temperature. Some animals are born with their eyes closed—others with the eyes open ; the former, Dr. E. observed to be those whose temperature fell when removed from their parents—the latter, those in which it kept steady; or, in other words, the former were cold-blooded at birth, the latter were warm-blooded. In confirmation of this, he also noticed that the heat, or rather the power of preserving heat, of the former, rose gradually to the standard of the adult animals, and at the end of a fortnight, which is the period when the eyes open, it reaches it perfectly.

"Thus (says he) the state of the eyes, though having no immediate connexion with the production of heat, may yet coincide with an internal structure influencing that function, and certainly furnishes signs which serve to indicate a remarkable change in this respect, since at the period of the opening of their eyes, all young mammalia have nearly the same temperature as adults." 70.

The author does not so cleverly point out any external differences in the young of the warm-blooded and of the cold-blooded birds. It is stated, indeed, that "the young birds, which are able to run about and preserve their temperature, are only covered with a tolerably thick down, and not with feathers;" but the same may be said of the others; and most annoyingly are we informed, in the very next sentence, that "the mammalia born with closed eyes, and birds *hatched without feathers*, produce so little heat, as to be, in relation to the air, in the state of cold-blooded animals" !! How are we to reconcile these two? But even in the case of the young of the warm-blooded birds and mammalia, there is this difference between them and the adult animals, that although the heat of the former is as high, or nearly so, and although they have the power of retaining it, when exposed to ordinary temperatures of the air, yet, if the temperature is much reduced, say to 36° or 40°, they are found to be cooled more rapidly and to a greater extent. This was easily proved by introducing them into glass vessels, surrounded with a mixture of salt and ice. We are, therefore, warranted to make the general conclusion, "that the power of producing heat, in warm-blooded animals, is at its minimum at birth, and increases successively to adult age." On this principle, we may, perhaps, account for the very great mortality among new-born children in Paris, especially during the Winter months.

"It is the custom in France to convey infants, within a few hours of their birth, to the office of the mayor of the quarter in which the nativity took place, in order that the birth may be registered, and the child become possessed of its civil rights. A careful comparison of the register of births, with the register of deaths, furnished statistical observations on so large a scale, that there can be no room to doubt the correctness of the results. It appeared that the proportion of deaths, within a very limited period after birth, compared with the total births, was much greater in winter than in summer, and that this difference of proportion, was much greater in the northern and colder departments, than in the southern and warmer. The details of this investigation are recorded in a paper which the Doctors have presented to the Institute. They have since continued the inquiry, and the following extract from a letter which I have received from Dr. Milne Edwards, will shew the accordance of their results.

'In order to ascertain in a more positive manner than before, whether the mortality of new born children is increased by their being carried to the *maire* immediately after birth, we obtained from the minister of the interior, necessary orders to have the tables of mortality of infants made in a certain number of parishes, where the inhabitants are scattered over a larger surface of ground; and in others, where they are, on the contrary, agglomerated around the *maire*. It appeared evident to us, that if our opinion was correct, the increase of mortality during Winters, must be much greater in the former parishes than in the latter, and such is, indeed, the result actually afforded by our tables.'" 476.

We come now to the consideration of the heat of adult warm-blooded animals, and to the diversities and modifications which different animals exhibit in their power of producing, and consequently of retaining their heat; and first, of that singular tribe which hibernate at particular seasons. It is well known that some animals, on the approach of Winter, fall into a state of torpor, and death-like inactivity, and remain so till the return of warm Spring-weather; their temperature all the while being scarcely above that of the surrounding atmosphere; their respiration is feeble, irregular, and occurs only at long intervals, and nutrition, at least from without, entirely ceases. The species best known in this climate are the bat, hedgehog, marmot, and several sorts of dormice.

What is the true cause of hibernation? An experiment or two will be sufficient to determine this point. It is necessary, however, to premise, that Buffon was quite mistaken, when he alleged, that the heat of hibernating animals, during their state of active life in Summer, was only 54°; for in truth, they do not differ in this respect from other adult mammalia; Spallanzani, Hunter, and others, found that their temperature was from 93° to 98°. It was ascertained, however, by M. de Saissy, that their temperature falls gradually as the season advances beyond the month of August, at which time it is probably at its maximum.

"On the 6th of August, the temperature of the air being 73° Fahr. that of a marmot was almost 98° Fahr. in the axilla. On the 23d of September, the air was 64° 5′ Fahr. and the marmot 88° Fahr. and on the 10th of November, the air was 44° 6′ Fahr. and the animal only 81° Fahr. which is 16° 6′ Fahr. lower than its temperature in the month of August. A garden dormouse, examined under the same circumstances had a warmth of 88° Fahr. on the 3d of August; 87° Fahr. on the 23d of September; and only 69° 8′ Fahr. on the 10th of November, having lost 28° Fahr. since the first examination. The examination of a hedgehog gave the following results. It was 99° Fahr. on the 3d of August; 96° Fahr. in September; and only 57° Fahr. having lost more than 40° Fahr." 77.

Reasoning upon these facts, Dr. Edwards was led to suspect, that hibernating animals, although they may exhibit, in their active state, as high a temperature as other mammalia, have not the same power of resisting cold; and to determine this point, he instituted the following experiment.

"In April, 1819, the air being 61° Fahr. an adult bat, of the long-eared species, recently taken, in good condition, and at the temperature 93° Fahr. was placed in an earthen vessel, which was cooled by a mixture of ice and salt, which surrounded it, till the air within was reduced to 33° 8′ F. It had a cover, which allowed a free communication with the external air. After the animal had been there for an hour, its temperature was reduced to 57° F., being a loss in this short space of time of 36° F. Guinea-pigs, and adult birds, placed in the same circumstances, lost, at the utmost, no more than two or three degrees, although the influence of the cold was prolonged in this case, to compensate for the difference of size. We see from this, that bats are in the habit of producing less heat than animals which do not hibernate; and it is to this cause that we must chiefly ascribe the reduction of their temperature during the cold season." 79.

We have now only one step further to advance in our enquiries, after the true cause of hibernation. The following observation of Saissy gratifies our wishes. In the months of May and June, he enclosed a marmot with a little straw, in a copper box, the lid of which—

"Was pierced with a hole about half an inch in diameter. After leaving it in an ice-house for twenty-four hours, he exposed it to an artificial cold of 10° c. or 18° F. below the freezing point. It fell into a profound torpor eleven hours after, and although its temperature had fallen from 35° c. or 95° F. to 5° c. or 41° F.; yet its health did not appear more altered than in the ordinary circumstances of hibernation; for, on being afterwards exposed to the warmth of the atmosphere, it recovered from its torpor, and resumed its wonted activity.

This interesting fact shews that hibernating animals may become torpid at any season, and from other causes than the want of nourishment; and also that the phenomena which take place in hibernation do not proceed from any change in the organization of the animal at the end of summer, as some have supposed. We may now compare the hibernating with the other mammalia, with reference to the external temperature during spring and summer, when both are in the enjoyment of the full activity and vigour of life.

In the preceding experiment, the degree and duration of the cold to which the marmot was subjected, were such as might lead us to question whether non-hibernating animals would not have lost as much heat under the same circumstances, enclosed in a box with so small an aperture, that respiration might have been impeded. We may also conclude, from the following observation of the same author, that cold was not the only influential cause :—'The marmot which I reduced to a torpid state at two different times, only became so, I believe, in consequence of its occurring to me, to close the aperture in the lid at a time when its respiration was much enfeebled. It was only in this manner that I succeeded, for all my previous attempts had been vain.' We have here, then, a combination of two causes—external cold, and diminished respiration; without being able to distinguish the respective effects of each." 79.

The general conclusion to which Dr. Edwards arrives, is, that hibernation is mainly to be attributed to a deficiency in the power of producing heat, in consequence of which they are incapacitated from resisting the external cold, in Winter. It is very generally believed, that all other animals maintain a steady and uniform temperature during all seasons; but we shall find that this idea is not strictly correct; and in truth there is no strongly drawn line of demarcation between the hibernating, and many other families of the mammalia. Perhaps no part of Dr. Edwards' researches is more interesting than the discovery (supposing that future experimenters confirm his statements) of the influence of the seasons on the generation of animal heat. We have already seen that frogs, and other batrachia are capable of withstanding cold much more energetically in Winter than in Summer, and that this difference is in all probability attributable, to their bodies being then more powerfully calorific; the same holds good of the higher, or warm-blooded animals, including birds and the mammalia. Dr. Edwards, in the month of February, took five sparrows, and introduced them into a glass vessel, in which the air was cooled to the freezing point; they were kept in for three hours, and when examined by a thermometer, it was ascertained that the average loss of heat in that time was only 7° Fahr. The same experiment was performed in July, and at the end of the third hour, the average loss amounted to 10° Fahr.; in August, to $8\frac{3}{4}$° F.; it appears, therefore, that these sparrows did not resist the cold so well in the Summer as in the Winter; and we are led by analogy (for no direct experiments are detailed) to the belief, that it is a general law of animal existence, that the power of producing heat varies with the season; that it is at its minimum point in July, and at its maximum in January, or December. It is proper, however, to mention, that we have been somewhat puzzled to understand how the power of "producing heat" can be at its minimum in the middle of Summer, if the actual temperature of the animal is discovered to be higher. Compare the following two passages;—at page 127, it is stated—"the power of producing heat is in Summer reduced to its minimum;" and at page 256, we are informed, that the averages of the temperature of sparrows and yellow-hammers ran progressively—

"From the depth of winter to the height of Summer, within the limits of two or three degrees cent. The observations upon sparrows gave me the greatest difference. The average was in February 105°·4 Fahr.; in April 107°·6 Fahr.; in July 110°·78 Fahr. I afterwards noticed the contrary course in the decline of the year.

Hence I judged, that man also would experience variations of temperature under the influence of the seasons and of the change of climate, if not to the same extent, at least within appreciable limits. Dr. John Davy, on his return from Ceylon, informed me, that the temperature of the inhabitants of that island was higher than ours by one or two degrees of Fahrenheit, and that he had observed a similar change in the same individuals before their departure and after their arrival." 257.

We need a commentary on these passages; Dr. Marshall Hall would account for the greater energy which animals possess, during Winter, of resisting cold, by referring it to diminished irritability.

If we attend to the habits and instincts of animals we shall observe many curious illustrations of this law, and also of the remark which we made above, that there are many links of connexion between the strictly hibernating animals, and those whose temperature is most steady and uniform; thus, for instance, mice, if exposed to a moderate cold in Winter are surprisingly benumbed; hence their practice of making nests, for the sake of warmth. It is by no means improbable, that even different individuals of the same species, say, for example, of mankind, vary considerably in their powers of generating heat, and consequently of withstanding any great reductions of external temperature. Unwilling to leave this subject till we have put our readers in possession of whatever is interesting and valuable in the work before us, we shall now enquire, whether any mutual connexion can be traced between the production of heat and any other function of the animal system, before proceeding to treat of asphyxia and the modifications of respiration, by age, season, and so forth. According to the plan which we have hitherto pursued, we shall propound some experimental data, and thence deduce our inferences. M. de Saissy brought a bat, which was profoundly torpid, and whose temperature was 39° F. into a room where the temperature of the air was only 28° F.; he irritated it mechanically, and left it; it awoke in an hour; thirty minutes after, its heat had risen to 59°, and in another half hour to 80°.

Again, a hedgehog, equally benumbed, and whose temperature was 37°, was treated in a similar way; it awoke in two hours, when the heat was found to be 54½°, and in an hour after 86°. Similar experiments were performed on dormice with similar results. If, instead of employing any mechanical stimulus to wake the animals, we merely expose them, while torpid, to a very considerable cold, the effects were nearly the same. A dormouse, whose heat was 39°, was placed in a window, exposed to a stream of very cold air, when the thermometer stood 7° below freezing; it awoke in about an hour, and ran into its cage with agility; its temperature gradually rose to 97°. If the cold be continued too long, all the animals, viz. marmots, dormice, and hedgehogs, relapse into a fatal lethargy. What are the inferences to be drawn from these experiments? It is evident that the recovery of heat was not caused by the medium in which they were placed, since the temperature of the air was lower than that of their bodies, and that therefore it must have been a vital, not a mechanical process. It was observed, both by M. de Saissy, and by Dr. Edwards, that the respiratory movements were at first very feeble and scarcely perceptible, but increased progressively to the degree of rapidity and extent which they have, in the active state of existence; we may therefore presume that there is a mutual connexion and dependence of the two functions of respiration and production of animal heat. Having thus largely treated of some of the more interesting phenomena of the latter function, our next enquiry is to similarly investigate the former in warm-blooded animals. Dr. Edwards directs our attention in the first place, to the circumstance of different individuals of the same species, size, and age, requiring different quantities of air for the continuance of life in a given period; we shall state the experiments in his own words, to avoid all fallacy.

"I placed some sparrows, in every respect as much alike as possible, in vessels of the same form, and containing each a litre, or about a pint and three quarters of air, inverted over mercury. Still further to ensure equality, in the condition of the experiments, I performed them simultaneously, thereby avoiding differences in the temperature, pressure, and humidity of the air. In a great number of experiments, I ascertained that there was sometimes a considerable difference in the duration of life with the same quantity of air, and that the shortest and the longest duration might differ by one-third. The air, however, was altered nearly to the same degree by all, so that the duration of life was principally affected by the comparative rapidity in the consumption of oxygen." 93.

But not only do different individuals vary in this respect, but the same individuals, we have grounds to believe, at different ages. It is not easy, indeed, to experiment on the same animal at periods much remote from each other, but we may employ young and adult animals of the same species, and make allowance for differences of size. Dr. Edwards, acting on this view, took some young, and some full-grown sparrows, and confined them in vessels which contained an equal quantity of air, and he found that the mean duration of the life of the former was more than 14 hours, while that of the latter did not exceed an hour and a half; a difference quite disproportionate to the difference in their bulks, and consequently in the capacity of their lungs; he therefore inferred, that a young sparrow vitiates proportionately less air than an adult one.

The experiments were repeated frequently on sparrows, having a less disparity in age and size; and the results proved to be always in accordance with the above law. Our readers will recollect that it has been already shewn, that young animals generate less heat than adults; and to this we may now add, that they consume less oxygen during respiration, even although their breathing be quicker, than in after-age. Let us advance another step, and enquire, do different species of animals vitiate different quantities of air; or, in other words, do similar quantities of air support the lives of different species, *cæteris paribus,* for different periods of time? Dr. Edwards placed in vessels, containing equal quantities of air, puppies a day or two old, and guinea-pigs about a fortnight old.

"The dogs were taken out at the end of four hours and fifty-nine minutes, and the Guinea pigs after an hour and forty-two minutes. By analysis of the air, the mean of the quantities of oxygen was found to be sensibly the same. Regarding only the difference of bulk, the consumption of air by the puppies ought to have been much more rapid, because they were larger, but when we recollect the fact previously established, that young puppies at birth produce much less heat than Guinea pigs, we see here the same relation to exist between the consumption of air and the production of heat, that we had determined in the case of the sparrows." 96.

Another important question remains:—Does the change of seasons occasion any modifications in the constitution of warm-blooded, as of cold-blooded animals; so that, supposing the quantity, temperature, and density of the air to be the same, it shall be consumed by them in different proportions at different periods? Analogy would lead us to answer it in the affirmative, and Dr. Edwards assures us that experiment proves it so. He found that, in January, six yellow-hammers lived in a certain quantity of air, of the temperature 68°, for 1 hour, 2½ minutes; and in August and September, every thing being alike, for 1 hour, 22 minutes. We need not relate more of the experiments, as they all lead to the same result, viz. that warm-blooded animals vitiate the air less quickly in Summer than in Winter; and our readers will, no doubt, recollect that it has been already shewn, that they also generate less heat; the seasons, therefore, it would seem, exert considerable influence on the function of respiration.

The analysis of Dr. Edwards' researches and opinions, which it has been our endeavour to exhibit in the preceding pages, cannot fail to attract the attentive study of all who feel an interest in animal physiology; it is beautiful to trace the mysteries of life through the different classes of animals, and it is, in truth, a pleasing reward to our labours, when we can arrive at the discovery of any simple, uniform, and all-pervading laws which regulate their functions. No one has worked more zealously and with greater success, in this field of enquiry, than our author; and, as his researches are both original and important, we have felt a pleasure in widely developing them, for the benefit of all our professional brethren. We have not strictly followed the chapters and sections of the work in detail, as they have appeared to us to be frequently disjointed and confused, but have rather brought together, from the different parts, all such details as had reference to, and illustrated, the particular question under consideration. Several subjects of great interest still remain; and first is that of *Asphyxia* in warm-blooded animals.

So essentially necessary is the function of respiration, that it is well known that life is apparently extinguished in three or four minutes, if any adult animal, no matter however large or small, be deprived of air; we have said "apparently," because organic life continues after all external motion and sensation have ceased; but even this lasts only for a very short time longer, so that the apparent death of an animal so treated is scarcely short of the actual death. The chief object which Dr. Edwards had in view, in his researches on asphyxia, was to endeavour to find out, whether the duration of life of warm-blooded animals, when immersed in water, is steady and uniform in all species, and at all seasons, and at every period of age; or whether it varies according to these varying circumstances and conditions, in the same manner as the power of producing heat has been found to do. From reasoning alone on the preceding details, we should expect certainly that considerable diversity would exist, for we have already discovered that warm-blooded animals do not consume the same quantity of air, and that they differ from each other, in this respect, according to varieties of constitution, of age, of species, and also according to the temperature of the atmosphere; analogy, therefore, might lead us to infer, that those animals which could live longest in a limited quantity of air, should be less speedily asphyxiated by drowning; and we shall now find that experiment confirms the analogy in most particulars. It is necessary to premise that, when it is said that some animals live longer than others under water, we allude to the retention of organic, and not of animal vitality; for Dr. Edwards, at page 133, distinctly states, that "it is remarkable all voluntary motion, and likewise sensation, are lost in a very short time, say three or four minutes, even in individuals which live the longest in asphyxia." A reader of the present work may very easily be misled, by the vague and indefinite language which is employed at different parts: thus, where it is stated at page 87, &c. that drowned animals gave signs of life for half an hour or longer, he might suppose that voluntary movements continued all this time; but such, he will now understand, is really not the meaning. With this explanation, we proceed to ascertain the different modifications of asphyxia, and first in respect of age. It is probably known to most, that the ingenious naturalist, Buffon, was led by some experiments to believe that many, and perhaps all, mammiferous animals might be made truly amphibious, if subjected, immediately after birth, to a peculiar management of alternate withdrawal from, and exposure to, the air.

"He placed a greyhound bitch of the largest species, when on the point of giving birth to young, in a tub of warm water, and secured her in such a manner, that she was obliged to bring them forth under water. These were afterwards for the sake of nourishment transferred to a smaller tub of warm milk, but without giving them time to breathe. They remained there for above half an hour, after which they were taken out and all found alive. They began to breathe, which they were allowed to do for half an hour, and were then again plunged in the milk, which had been warmed again in the mean time. There they remained for another half hour, and when they were again taken out, two were quite strong, and seemed not to have at all suffered. The third appeared drooping; but was carried to its mother, and soon recovered. The experiment was continued on the other two, they were allowed to breathe a second time for about an hour; and were then plunged once more in the warm milk for half an hour, after which they appeared as strong as before. However, being taken to their mother, one of them died the same day, whether by accident or from having suffered from the privation of air could not be ascertained. The other lived as well as the first, and both thrived as well as the other puppies produced after the bitch was removed from the water, and which had not undergone the ordeal."

"I have not," he says, "pursued these experiments further, but I have seen enough in them to persuade me that respiration is not so absolutely necessary to the new-born animal as to the adult, and that it might perhaps be possible, by proceeding carefully, to prevent, by these means, the closure of the *foramen*

*ovale,* and produce excellent divers; and species of amphibious animals, equally capable of living in air and in water." 135.

The facts are no doubt true—the inferences are certainly not. Buffon was not aware of the distinction of animal from organic life; he was not aware that, although vitality continued for a certain time, all voluntary power and all consciousness had long been lost. As the puppies were in milk, he was prevented from seeing the phenomena which they presented—he was deceived by the facility with which they were restored to life. But it would be of little advantage to an animal intended for diving, merely to be able to remain a long time under water, if it had not the use of its senses and of voluntary motion all the while. M. Legallois performed numerous experiments of a similar nature as the above, and with like results: he found that rabbits, when just born, lived for about 28 minutes under water; when 5 days old, for 16; when 10 days old for 5½; and when 15 days old, they appeared to have no superiority over the adult animal. Kittens also present similar phenomena; but all animals do not. Dr. Edwards found that a guinea-pig, at birth, when plunged in water, lived only three or four minutes longer than the adult; thus distinctly proving, that different species of young animals possess different powers of resisting asphyxia, and that those animals which generate most heat resist it most feebly, adding a fresh argument to the position which we have repeatedly declared, that the organic vitality is, *cæteris paribus,* inversely as the energy of the calorific function.

Of all the mammalia, the hibernating animals are those which can live longest without the contact of air; but this superiority exists only as long as they are torpid; for Dr. Edwards found that bats, in Summer, were asphyxiated by drowning as speedily as other animals; during their torpor, they may be kept in carbonic acid for upwards of four hours, without appearing to suffer from the experiment. Passing now to the modifications which differences of temperature exert on the slowness of asphyxia, the same general laws are discovered to be applicable here as on former occasions. Kittens, if drowned in water cooled to freezing, ceased to give signs of sensibility and motion in 4½ minutes; if the water was 50°, in 10½ minutes; if 68°, in 38¾ minutes; if 86°, in 29 minutes; if 104°, in 10½ minutes. Similar experiments on puppies gave the same results. These observations of Dr. Edwards are most satisfactorily confirmed by the details of some experiments, which, it appears, Sir Astley Cooper performed on puppies and kittens, as far back as 1790. We shall select four of these, as they serve to illustrate this very interesting subject.

"Experiment I.—A young puppy was immersed in warm water, at about 120° Fahr. for one minute and a half. It struggled violently, and during the latter part of this time threw out the air from its lungs. It then remained still for another minute and a half, when its struggles were renewed, at which time it voided its excrement. These efforts were soon over. After remaining still for three minutes, it was put into another vessel containing water of the same temperature. In this it gasped twice or thrice. In ten minutes after its first immersion I opened it—a slight undulation was observable at the lower part of the right auricle of the heart, and there was some motion in the intestinal canal. The action of both ceased in about a minute, and could not be reproduced by the irritation of touching and piercing it. Thus, then, the action of the heart was destroyed eleven minutes after its first immersion.

Experiment II.—A puppy, of the same age as the last, was immersed in water, at about 56° Fahr. For one minute and a half its voluntary motions continued violent—it expired the air from the lungs, and then was quiet. At the end of another minute and a half its struggles were renewed. Its excretions passed off. For two or three minutes after it continued to gasp. It was then thrown into another vessel containing water of the same heat. It gasped at the end of every minute, and ten minutes after its first immersion it was opened. The heart acted vigorously, and there was strong peristaltic motion in the intestines. The action of the heart continued strong for nineteen minutes after it was opened, when it began to undulate—it undulated for four minutes, when all action ceased. At the end of twenty-nine minutes, then, the heart of this animal acted as strongly as that of the first, which died in ten minutes."

"Experiment V.—A kitten was put into cold water, and after about two minutes ceased to struggle. For some minutes after it had convulsive motions. Twelve minutes after it was immersed, its abdomen and thorax were opened. The heart was contracting, and the intestines moving. The heart continued to do so for half-an-hour.

Experiment VI.—A kitten was immersed in water heated to 100° Fahr. Its efforts seemed rather more violent than those of the other in the fifth experiment. Its convulsions were sooner discontinued. Twelve minutes after its immersion it was opened—no action could be observed, either in the heart or intestines, nor could it be produced by stimuli or irritation." 473.

To illustrate still further the subject of the influence of temperature on asphyxia, we shall detail an experiment of Dr. Edwards, from which it appears that, if the heat of an animal be reduced previously to immersing it in water, death is not so quickly induced. He took some young sparrows, in the month of May, when the thermometer stood at 61°, and cooled them artificially.

"One of them, which had, in half an hour, cooled from 35° cent. to 19° cent. or 95° to 66° Fahr. was afterwards warmed, and when he had regained his original heat was immersed in water; he lived in it eight minutes; now adults live only a minute and a few seconds. The other, whose temperature was only reduced to 26° cent. or 79° Fahr. was in like manner warmed again and immersed in water; he gave signs of life for only four minutes. Others, which lost little heat by exposure to the air, differed equally little from adults in the duration of life under water." 88.

Before quitting the theme of asphyxia, we wish to direct the reader's attention to one phenomenon connected with it, viz. that of the continuance of the circulation when all sensibility and motion have ceased. Bichat was the first who fully illustrated the subject, and pointed out to us that the dark venous blood circulating in the arteries acted as a sedative on the nervous system, and, consequently, on the functions, among which is respiration, dependent upon it. He did not, however, ascertain whether the organic muscular life was kept up by the circulation of dark blood, for a longer time than when such circulation is prevented. Dr. Edwards had found that, in the case of the batrachia, the action of the nervous and muscular systems remained 20 hours longer under the former, than under the latter circumstances; he drowned some frogs whose hearts had been removed, and an equal number which had not been mutilated, in water of the same temperature; the results, in all cases, exhibited a marked difference in favour of the latter. Similar experiments were performed on kittens; and it appeared that, when the heart was excised, and the circulation thereby stopped, drowning caused muscular death in a quarter of an hour; whereas, others, on whom no injury had been inflicted, gave signs of life for about half an hour. We cannot, therefore, now doubt that the circulation of venous blood contributes somewhat to maintain the life of all animals after the cessation of external motion, and during that state which we call apparent death. The duration of this latter state is affected by the temperature of the water in which the animal is immersed, as is shewn by the following experiments.

"Towards the end of December, the previous temperature having been very low, the hearts were taken from eight frogs. Four were placed in water at 68° Fahr., and four others in water at the freezing point. The first set lived on an average an hour and three minutes, the second eight hours and fifty-five minutes. Temperature, therefore, acts upon the frogs when circulation is destroyed, and which are, as it were, reduced to the exclusive action of the nervous system, in the same manner as in those whose circulation is in full activity. The hearts were cut out of three new-born kittens; one was put in water at 68° Fahr., another in water at 104° Fahr., and the third in water at 32° Fahr. The first lived thirteen minutes and thirty seconds; the second seven minutes; and the third five minutes. Now these animals lived only by the nervous and muscular systems, and if we compare the result of these experiments with those related in Part III. Chap. IV. performed on animals of the same species, whose circulation was not destroyed, and which were placed in the same circumstances, it will be seen that the temperature exercised upon both an analogous influence. For it was in water at 68° Fahr. that they lived the longest, much shorter at 104° F. and the shortest at 32° Fahr. Temperature, therefore, within these limits, exerts a direct influence on the vitality of the nervous system." 140.

The subject which next demands our attention, is that of perspiration. Our readers doubtless know, that to Sanctorius we are indebted for the original researches and experiments to determine the actual and proportional amount of this secretion. From numerous calculations he estimated, that five-eighths of our ingesta escape in the form of invisible transpiration. Dr. Edwards has pursued this enquiry with much diligence; he states that, if you keep an animal in any vessel, and compare its weight at intervals of two, six, or nine hours, you will find that there is a uniform and progressive diminution of the loss by perspiration; and that the amount of the loss varies exceedingly, according as the atmosphere is dry or moist; in dry air, of a moderate temperature, the perspiration was ascertained to be six times greater than in humid air. This is, indeed, contrary to the vulgar opinion, which has arisen from observing, that there is generally an accumulation of moisture on the skin when the weather is damp; but the true cause of this is to be found, not in any increased perspiration, but in diminished evaporation. The quantity of perspiration is much influenced by a variety of contingencies, as, for example, 1st, by digestion. Sanctorius has asserted, that perspiration is very slight for three hours after a meal; but Keil, Dodart, and others, contradict it; and Dr. Edwards is inclined to believe that the quantity is scarcely at all affected. 2d. By sleep, which has generally the effect of increasing perspiration, both in health and disease; but this effect is modified, according as the stomach is idle or engaged. "Qui vacuo ventriculo it cubitum, eâ nocte tertiam partem minus more solito circiter perspirat;" and, "Horâ dormitionis meridionæ a cibo corpora aliquando libram aliquando selibram excrementorum occulte perspirabilium excernere solent," are two aphorisms of Sanctorius, and they are confirmed by the experience of every one. 3d. By the dryness or humidity of the atmosphere, as already stated. 4th. By the motion, or rest of the air. Sanctorius and Gorter supposed that the perspiration was diminished by windy weather; but this conclusion was not the result of experiment but of conjecture, and is amply refuted by Dr. Edwards, who has found, that the motion of the air uniformly tends to increase insensible perspiration. 5th. By variations in the pressure of the atmosphere; the amount of perspiration being inversely as the pressure. 6th. By temperature; but here we must observe, that in all discussions on the subject of the functions of the skin, it is necessary that we should correctly and definitively distinguish insensible perspiration from sweating or transudation; the one is a physical, the other is a vital process; the one is regulated by the laws of evaporation, and is a consequence of that porosity of organized bodies, by which liquids, when near surfaces in contact with the air, diminish in quantity, by being converted into vapour, even though the pores be such as not to give passage to a single drop of fluid; the other is as much a secretion as the salivary, and the urinary discharges; whenever the one is increased, the other is diminished; and by keeping the law in remembrance, we shall readily be able to understand, how the insensible perspiration may be much augmented, and yet the skin be dry, and we shall have no difficulty in answering a question which has been started—"is perspiration susceptible of being suppressed?" in the negative. We cannot, in any circumstances, check the insensible and sensible perspiration at the same time; if the air be dry and cold, although the latter appears not, the former continues; and if, on the other hand, it be warm and moist, we know well, that sweat bursts out at every pore.

"We ought, therefore," says Dr. E. "to be careful how we take literally what we find in medical books respecting suppressed perspiration. There can be no such thing. That there may be suppression of sweat, is evident to every one; but it does not follow that even in these cases there is no transudation." 175.

*On Absorption by the Skin.* Although there has been much discrepancy of opinion upon this theme, authors have limited their examinations and experiments too much to one animal, or one set of animals, and have neglected others in the scale of creation, which, if explored, might have tended, most likely, to have facilitated a correct determination of the question. Our readers will remember, that in the commencement of the review of Dr. Edwards' work, we alluded to some observations of the author, on the absorption of fluids by the skin, in frogs and fishes; the phenomena are too obvious to be gainsayed by any one, and the results may be even appreciated by an ordinary balance. As the subject is one of interest, we shall narrate an experiment which the Doctor performed on a lizard, whose skin is entirely scaly, and therefore very unfavourable to rapid absorption.

"It was exposed to the open air in order to remove it from the point of saturation and cause a sensible loss of weight. Perspiration in these animals being very slight, several days were allowed. It was then introduced into a tube and fastened by a fore and a hind foot. I then placed it in water so as to immerse only the tail, the hind legs, and the hinder part of the trunk. It was afterwards weighed at distant intervals, and found to have successively increased in weight, until it had supplied the loss incurred by perspiration in the air. The experiment was then stopped. This absorption was not mere imbibition limited to the surface; the water penetrated deeper, and was distributed through the system. The body and the limbs had resumed their roundness and plumpness, and life, which would, ere long, have been extinguished in the air, as had happened to several other individuals, which were exposed at the same time, was prolonged by the liquid which absorption at the external surface had furnished to repair the loss which had been sustained." 182.

Dr. Edwards is a strong advocate in favour of the absorbing powers of the skin in all animals, and as he maintains that transudation goes on even when the body is immersed in water, he alludes to the very experiments which M. Seguin has brought forward to prove non-absorption, as confirmatory of his views. We regret that the experiments on which he founds the premises have not been detailed; whether this omission is chargeable to the author, or to his translators, we cannot say; but we should advise Dr. Hodgkin to revise Chap. VI. of Part I.; it is most inconclusive and unsatisfactory; the inferences appear to be drawn from vague assertion, not from exact observation. We shall, however, transcribe the following passage, as it displays the chain of reasoning employed by Dr. Edwards.

"If the diminution of the weight of a man plunged in a bath be exactly equal to the loss incurred by pulmonary perspiration, the absorption by the skin is equal to the transudation by the same organ.

The proportion, as quoted in the former chapter, from Lavoisier and Seguin, which pulmonary perspiration bears to general perspiration in the air, is 6° to 18°; in their second Memoir on Perspiration (Annales de Chim. vol. xc. p. 22.) they give it 7° to 18°. In the first series of experiments, in which Seguin compares the loss in water to that in air, he finds the proportion 6°·5′ to 17°. Hence it follows, that the loss in the bath did not exceed the pulmonary perspiration. No more is necessary to render this case an example of absorption, since we do not find in it the excess of loss which would result from cutaneous transudation. Seguin, however, infers from it the absence of both functions; and this explanation will equally account for the result; but his own researches oblige him to admit transudation in water at higher temperatures, although he maintains, that between the degrees of 54°·5′ and 72°·5′ Fahr. neither transudation nor absorption takes place." 183.

That transudation most assuredly takes place in water, which has been heated considerably, is proved by many; thus—

"Lemonnier, after staying eight minutes in a water bath at 45° cent. or 113° Fahr. lost twenty ounces, which is at least double that which Delaroche and Berger lost at the same temperature in a vapour-bath, and at a temperature of above 90° cent. or 194° Fahr. in dry air." 200.

And from many most cautious trials, the conclusion has been drawn by Dr. Edwards, that, other circumstances being equal, as regards the skin, "that state of air which has the greater heating power, will occasion the more copious sweating." Now, as steam has a greater capacity for caloric, than atmospheric air, a vapour bath of a given temperature will be more powerfully sudorific, than one of heated air; and reasoning by analogy, we infer, that a hot water bath will exert a still greater effect in *promoting* transudation.

*On the Uniformity of Animal Heat.* Although every professional man is acquainted with the very extraordinary power which living bodies have of preserving their temperature nearly steady, yet we deem it may not be unacceptable to our readers to mention a few of the most striking examples. Duhamel relates, that a baker's daughter remained 12 minutes in an oven heated to about 264°, without much inconvenience. Blagden and Fordyce repeated the experiments, with like results. Delaroche and Berger extended their observations to the lower animals, and ascertained that the effects varied, not only with the temperature, but also with the state of the medium, as to dryness, or humidity.

"They introduced into a stove heated to 110° a cat, a rabbit, a pigeon, a yellow-hammer, and a large frog; the greater number at first remained undisturbed, but in about half an hour they became agitated and their respiration was progressively accelerated for about three quarters of an hour, until it became panting. There was then a remission of the symptoms in almost all the animals. They remained an hour and a half; when none of them came out in a natural state, but in half an hour or an hour they appeared perfectly recovered.

It would appear, from these experiments, that vertebrated animals exposed to a dry and hot air of 45° cent. or 112° Fahr. are near the limit at which they cannot long survive. Indeed, the same individuals, after having sensibly recovered from the effects of the previous heat, were introduced into a stove, the air of which, at first 56°·25′ cent. or 133°·25′ Fahr. arose towards the conclusion of the experiment to 65° cent. or 149° Fahr. All except the frog perished at various periods, from 24 minutes to 1 hour and 55 minutes." 192.

To ascertain the relative effects of heated watery vapour—

"M. Delaroche could not support, above ten minutes and a half, a vapour bath, which, at first, at 99°·5′ Fahr. rose, in eight minutes, to 124°·25′ Fahr. and afterwards fell one degree.

M. Berger was obliged in twelve minutes and a half to come out of a vapour bath, of which the temperature had risen from 106°·25 Fahr. to 128°·75 Fahr. He was weak and tottered on his legs, and was affected with vertigo. The weakness and thirst lasted the remainder of the day.

These gentlemen, however, supported for a considerably longer time, without much inconvenience, higher temperatures in dry air.

The peculiar sensation which resulted from the impression of heat was much more lively in the vapour bath, it was a sense of scalding. These experiments have been cited, because they are comparative, but not as indicating the extreme of heat, which man is capable of supporting in the vapour bath for that space of time. Joseph Acerbi relates, in his voyage to the North Cape, that the peasants of Finland can remain for above half an hour in a vapour bath, the temperature of which is raised to 158° or 167° Fahr." 194.

We need scarcely remark, that the heat of any liquid of the same temperature is still more insupportable. Dr. Edwards states—

"I have never seen batrachians which could live above two minutes in water, at 40° cent. or 104° Fahr. although I have taken the precaution of holding a part of the head out of the water, to allow the pulmonary respiration to continue; whilst individuals of the same species (frogs), have supported the heat of air charged with vapour at the same temperature above five hours.

Lemonnier, being at Barèges, plunged into the hottest spring, which was at 45° cent. or 113° Fahr. He could not remain in it above eight minutes. Violent agitation and giddiness forced him to come out." 194.

The assertion that living animal bodies preserve a uniform temperature, must be taken with some limitations; all experimenters agree that there is a slight elevation when they are exposed to very high heat; and the general inference is, that " man and warm-blooded animals, under the influence of excessive heat in a dry air, may experience during life an elevation of bodily temperature, of from 12° to 14° F.—a higher degree they cannot bear." With respect to the cold-blooded vertebrata, it might be expected that, as the temperature of these animals differs no more than from one to three or four degrees from that of the external air, throughout the various seasons of the year, it would continue to exhibit a similar conformity in higher degrees of heat.

" In the experiments of Delaroche and Berger, the highest temperature attained, at the period of death, by individuals of this species placed in the stove, was 105°·67 Fahr. which is within the limits of the bodily temperature of warm-blooded animals." 197.

It has been much disputed, to what cause are we to refer the power of animals of resisting the heating effects of an elevated temperature. Franklin ascribed it to the cooling agency of evaporation from the skin; and this idea is most beautifully confirmed by the recent experiments of Delaroche and Berger; they are so ingenious and novel, that we shall transcribe the detail of them at length.

" One of those porous vessels, called by the Spaniards alcarazas, which are susceptible of evaporation from the whole of their surface, was introduced by Delaroche and Berger into a stove, with two moistened sponges and a frog. The temperature of the vessel and of the sponges had been previously raised to that of warm-blooded animals, from 100°·6 Fahr. to 105°·67 Fahr. The temperature of the stove varied between 126°·5 Fahr. and 142°·25 Fahr. In a quarter of an hour, the vessel, the two sponges, and the animal, were nearly of the same temperature, not exceeding the limit of that of warm-blooded animals. They retained it pretty nearly two hours. This term is remarkable. In order to arrive at it, the vessel and the sponges, instead of being warmed, cooled about a degree; on the contrary, the temperature of the frog, which was at first 70°·25 Fahr. rose to 98°·9 Fahr. in the space of fifteen minutes, and remained stationary for the rest of the time, maintaining itself, like the alcarazas and the sponges, at from 27° Fahr. to 38°·7 Fahr. below that of the surrounding medium.

A greater difference is observed, according as the external temperature is higher, but at the same time, the term at which that of evaporating bodies becomes nearly stationary is a little higher, in conformity with this increase of heat in the air.

If we suppose that as long as respiration is performed by an animal, especially a warm-blooded animal, it preserves the power of producing heat, a difference will thence be inferred between its temperature and that of an inanimate body with an evaporating surface, exposed to an excessive heat, although their point of departure should be the same. This conclusion is justified by the results of experiments made by Delaroche and Berger. The evaporation in a rabbit exposed to air from 144°·5 Fahr. to 189°·5 Fahr., judging by the diminution of its weight, was quite as great as that of the alcarazaz; the final temperature, however, of the rabbit was superior by at least 4°·5 Fahr.

Thus the same general cause, viz., evaporation, would alone be sufficient to retain the temperature of animals and inorganized bodies below that of the external air, when the latter is excessive, that is, when it is above the bodily temperature of warm-blooded animals; but below this limit it would be incorrect to attribute to this cause, as is generally done, the real or supposed power of man and other warm-blooded animals, to maintain uniformity of temperature under the vicissitudes of seasons and climates." 201.

### ON THE INFLUENCE OF LIGHT ON ANIMAL BODIES.

If frog-spawn be placed in water, excluded from the light, it is found that the development of the tadpoles is either prevented altogether, or takes place most slowly and imperfectly. When, instead of the spawn, we try the same experiment on tadpoles themselves, the transformation from the state of fish to that of reptile is equally affected ; most of the tadpoles do not undergo any change, even though they remain plump and increase in bulk, shewing that it is not from deficiency of nourishment, but from mere absence of light, and such as are transformed, acquire their adult structure much later than naturally occurs. It will be remembered that it was stated formerly, that when tadpoles were deprived of atmospheric air, by keeping them constantly under the surface of water, several of them passed from their larval to their perfect state ; but, as others did not, it became an interesting question to ascertain which was the most potent obstructing agent, the exclusion of light, or the prevention of breathing ? Dr. Edwards has no hesitation in stating, that light is much more necessary than respiration, and he alludes to many experiments in confirmation : he is even inclined to suppose that the almost unique occurrence of such an organization, viz. the presence both of lungs and of gills, as exists in the "Proteus anguiformis." may be dependent on the peculiar circumstances of its abode, which is the subterraneous lakes of Carniola, where the absence of light is superadded to the low temperature of the water. We alluded to this before, and need not, therefore, enlarge upon it.

### ON THE ALTERATIONS IN THE AIR FROM RESPIRATION.

Dr. Edwards differs from many preceding authors in some particulars respecting the chemical changes which the air undergoes when inhaled into the chest. We shall allude briefly to them in succession. It has been much disputed whether the quantity of oxygen which disappears, is strictly equivalent to the amount contained in the carbonic acid formed. According to the researches of our author, it appears that there is a great variation in the proportions of oxygen which is absorbed, and of carbonic acid produced, according to the species of animal, age, season, and other particulars. In some experiments, the excess of the former was above one third part of the latter ; in others, it was so trifling as to be scarcely appreciable. If the conclusions of Dr. Edwards be hereafter confirmed, their importance will be most extensive and influential, not only as reconciling the discrepant statements of authors of acknowledged ability, but still more as developing a most curious and hitherto unexamined operation of vitality, which affects, perhaps, all functions, but more especially those of respiration and production of animal heat ; we allude to the diversified modification of their phenomena, according to accessory and contingent circumstances. The next question which Dr. Edwards examines is, whether there is any change of quantity of azote in respired air. In a vast number of experiments on new-born puppies and Guinea pigs, he ascertained that there is an increase in the quantity of this gas. The same results were obtained when the respiration of birds was tested. It is, however to be kept in mind, that the quantity varies exceedingly at different times and in different animals; and hence a long-continued and diversified train of research is necessary, before we can be authorised to propound any general assertions. So necessary is it to be on our guard, in all enquiries into the operations of life, that we shall frequently commit grievous errors, if we hazard even a single induction from a few experiments ; there is an apt illustration of this in the topic which we are now considering ; it has been stated, only a few lines above, that the quantity of azote was found augmented in respired air ; now this holds true at one season, and not at another. From October to February, or thereabouts, Dr. Edwards discovered that there was, in reality, a diminution, and, consequently, an absorption of azote ; his opinions are founded on a most extended suite of investigations on different animals. He says—

"If the difference between the inspired and expired azote had been quite irregular and confusedly scattered throughout the course of the experiments, no reliance could have been placed in them, but on the other hand, when we see these differences, however slight, constantly bearing for a considerable period in one direction, and then having an opposite tendency during an equal space of time, notwithstanding the identity of the mode of experiment, we cannot but conclude that a real change has taken place in the subject under investigation." 224.

Humboldt and Provençal, in their long series of experiments upon the respiration of fishes, have proved that these animals absorb a large quantity of azote. Spallanzani asserts the same of reptiles, and of many other animals. On the other hand, Bertholet, Nysten, and Dulong have most unequivocally found, on some occasions, an excess of this gas. How shall we reconcile these contrarieties? This is, perhaps, not so difficult as may at first be imagined; let us only suppose that there may be continually going on, in animal bodies, an absorption and an exhalation at the same time; and then the mere predominance of the one function over the other explains away all our difficulties. But let us proceed cautiously, and first ascertain whether any unequivocal mode of trial may be devised, to prove that azote is really exhaled from an animal :—Now all that we have to do, is to place an animal in an atmosphere of oxygen and hydrogen; Allen and Pepys acted on this plan, and they have most distinctly shewn that the exhalation of azote was frequently so great, as to exceed the bulk of the animal; and that there was also a considerable absorption of hydrogen. Here, says Dr. E. is a proof that two functions are performed at the same time, and is the key to the observation above made, that in different cases, according to diversified circumstances, we may find equality, excess, or diminution in the azote expired, when compared with that which has been inspired. As yet, we have not spoken of the probable manner in which the carbonic acid, which is thrown off from the lungs, is generated—whether it is evolved as a gazeous exhalation from the blood, or whether it results from the oxygen of the atmospheric air combining with free carbon, through the thin membranous coats of the vessels. A simple experiment determines this question :—If an animal be made to breathe a gas which does not contain any oxygen, and if more carbonic acid is found than can be accounted as being the residue which may exist in the lungs, at the time of subjecting the animal to the trial, we are forced to the conclusion, that the acid has been evolved in a gazeous form. Now let frogs (especially in Spring, when, as we formerly shewed, their irritability, or organic vitality was most energetic,) be confined in vessels containing hydrogen, they will be found to live for $8\frac{1}{2}$ hours, and upon examination the air will be found to contain carbonic acid in a much greater quantity than what the lungs could hold. If this experiment be performed in the warm weather of Summer, or of Autumn, the frogs will not live above one half the time ; another apt illustration of the doctrines previously unfolded. Similar results were obtained by introducing fishes (cyprinus aureus, or golden fish) into vessels containing pure hydrogen. Carbonic acid was readily detected; and Spallanzani's experiments assure us, that the same holds good of snails, and other mollusca. Dr. Edwards extended his observations to kittens, and other very young animals, and concludes that the principle is of universal application, and that the carbonic acid in expired air is due to exhalation, and that it proceeds wholly, or in part, from the blood. Vauquelin, Vogel, Brande, and Sir E. Home, have proved that it does exist in the mass of blood, and may be disengaged from it when the vessel which contains it is placed in hydrogen gas.

We have now brought to a close our analysis of the experimental researches of Dr. Edwards, on the influence of some of the most important physical agents on the life of man and other animals; but something still remains to be done; and we must enquire whether the additional and more exact knowledge we have obtained can be made available by the medical man in the prevention and cure of diseases. We are not, indeed, of that number, who estimate the value of physiological discoveries, *only* by their immediate and very obvious practical applicability; but would rather regard every step which is made in the investigation of the mysteries of living bodies, important, per se, as a fresh fact added to our knowledge, and also as tending to elucidate the working of the whole machine; if such be the case, it needs not the sagacity of a prophet to foretel, that just in proportion to the correctness of this knowledge must be our hopes of rectifying the machine when out of order, and of keeping it unharmed from losses and injuries. We have left ourselves little space for alluding to the application of the foregoing discoveries in detail; our remarks must therefore be brief and concentrated. Dr. Edwards is of opinion, that during natural sleep the faculty of generating heat is considerably impaired; and in this manner he explains the common observation, that a cold which is not inconvenient to us while awake, is hurtful if we are exposed to it during sleep, and how intermittent and other fevers so frequently arise in ships and camps from the night fogs. The following remark is exceedingly interesting, and offers a most ingenious hypothesis on what are vulgarly denominated "trances."

"Natural sleep, in many species of hibernating animals, merits the denomination of *lethargic sleep*, from the remarkable diminution of temperature, respiration, and circulation, as well as of the external motions and excitability of the senses. It differs in intensity according to individuals and species. We have shewn the modification of the constitution which has the greatest influence in this respect; so that it will be easily imagined, that changes of this kind may take place in man, which will render his sleep lethargic, which state, however, is not to be confounded with the effect of disease or accident, such as privation of air or exposure to noxious gases. Instances of the lethargic sleep here alluded to, are to be found in medical works, and are apt to be regarded as fabulous, but my own experience has convinced me, that such cases do occur." 248.

Dr. Edwards alludes to the propriety of clothing very young children more warmly than adults; their power of producing heat having been proved to be inferior. In some fevers, those which Torti has designated "febres intermittentes algidæ," this power appears to be so much weakened, that the patient dies in the cold stage, the system being unable to recover its loss of heat.

As a general rule, vapour baths are to be preferred to those of warm water, on the principle that the vivifying action of the air may be conjoined with the softening effects of the warmth on the skin. As a proof that the exclusion of the surface of the body from the contact of the air may be injurious, we need only refer to the cases of many persons experiencing a difficulty of breathing whenever they employ a warm water bath. The same reason explains the faintness which results from remaining too long in the bath; and it will be readily conceived that these effects will vary in different individuals, according as the pulmonary respiration has a greater or less extent.

The influence which rapid evaporation from the lungs and skin exerts on our feelings, is very strikingly exemplified in the reports of those who have ascended lofty mountains, or gone up in balloons; they almost always experience thirst, which is sometimes intolerable, even when it cannot be ascribed to the fatigue of exercise; it is only momentarily satisfied by large draughts of drink; but if the air becomes charged with moisture, this most painful symptom is at once relieved; the evaporation is lessened, and absorption goes on actively. The good effects of frequently sponging the skin, during fevers, and also at sea, when the supply of fresh water is scanty, are other proofs of the impotence of the cutaneous functions. Dr. Edwards remarks, that in many experiments in which animals were confined in rarified air, he observed that a disposition to vomit was induced. This accords with what we notice in dyspnœa from other causes. Whenever the respiration is impeded, as in acute or chronic pulmonary congestions, a disposition to vomiting is frequently induced; the community of the nervous supplies of the lungs and stomach may possibly account for this phenomenon. Whenever some persons enter a room, much heated in cold weather, they experience a painful sensation in the chest; this is chiefly attributable to the rapid evaporation from the surface of the air-cells; and the old custom of placing upon the stove a vessel containing water, is warranted by theory, as well as by practice.

We stated that children, who are born prematurely, have a very feeble power of producing heat; hence it becomes an object of great importance in attempting to rear such children, to compensate the deficiency by external means.

The researches of Dr. Edwards point out to us the importance of the skin as an accessory, and in some degree a vicarious organ of respiration: from being unencumbered with hair or feathers, in man, impressions made upon it are much more lively; and in this respect he has the advantage over all warm-blooded animals, even the hibernating; their skin is less accessible to the air; and hence no doubt the reason that an adult can seldom or never be resuscitated, when all external motion has ceased by drowning. Man, on the contrary, whose skin is bare, delicate, and sensible, may be re-animated by the action of the air, when he appears to have lost under water sense and motion. A great error is frequently committed by medical men in their attemps to restore life, in cases of asphyxia; we have seen how very hurtful a too great and long-continued external heat and a confined respiration are, when the energies of life are faint and low. If momentarily and very suddenly applied, good effects may indeed be obtained; thus, the mere immersion of a new-born child in warm water, is frequently a most efficacious means of re-animating it; but, as soon as signs of returning irritability and feeling are shewn, or if they be very tardy in appearing, we had better desist from a method which, if too much prolonged, may be fatal. If an animal is plunged in water of 104° F. its movements are much more forcible, but less numerous, than at inferior temperatures.

While reflecting upon these remarks of Dr. Edwards, we have been induced to question, in our own minds, the propriety of a long-continued application of external heat, in the form of steam or heated air, in that disease which has so much baffled alike physiological speculation and therapeutic aid. In no member of the nosological catalogue, is there such a remarkable diminution of animal temperature as in cholera; the true explanation of it may be obscure; but we think that most will agree with us in partly, at least, referring it to the state of the blood. We have no intention of indulging farther in speculation, especially as we deem it not improbable that Dr. Edwards himself may have directed his attention to the disease in Paris; certainly no man is so well fitted to elucidate some of its mysteries.

In concluding our review of Dr. Edwards' researches, it must appear quite unnecessary to detain our readers with any long-drawn eulogy; we have already expressed our opinion of his high merits; and, after the perusal of the preceding pages, all will be fully enabled to form their own estimate. It has rarely fallen to our lot to analyze any book, containing more original and interesing observations; and, if a feeling of regret can mix itself with the pleasure of our labours, it has been, that a foreign country and a foreign language have had the honor of its parentage. The different sections of the book were presented, in the form of separate papers or essays, to the Royal Academy of Sciences at Paris, and obtained for the author, although a stranger, the honor of the physiological prizes. Would that a similar institution, conducted on similar principles of enlightened liberality, and directed by men of minds as free of the love of pelf, and of the bigotry of obsoletism, arose to add dignity and honor to our native land! But we fear that the heads of the profession, or rather, we should say, the autocrats of our different institutions, are not the beings from whom we can expect such good; and we must trust to that spirit of generous intelligence which begins to animate the great bulk of our medical brethren, for better days and nobler doings. It was acutely observed to us, by one whose high and varied talents may yet make his name revered by the profession, that monarchy may be the best government for a nation, but that, assuredly, it is a republic alone which science and literature can ever acknowledge. No; we can no longer endure the petty thraldom of pride and sycophancy. Shall it be said that in Britain, the land of civil liberty and enlightenment, where the path of distinction is as open to the humblest commoner as to the proudest noble, a meaner bondage and a more galling despotism exist in the medical legislature, than is to be found at Petersburg or Constantinople? But the time is at hand, and a mighty change is awaiting us—for it has been well observed, that "the moment oppression passes one step beyond endurance, that step takes to liberty." May the day soon come, and may we have our Academy of Sciences, to foster and reward the professional talent of those, who now seek and find it elsewhere. Dr. Edwards probably felt that his labours would be better appreciated, and we may certainly add, more zealously examined, in Paris than in London; be this as it may, he has proved himself one of the most accomplished physiologists of modern times, and we trust that he may yet reap fresh and more distinguished laurels. His present work exhibits the most unwearied industry, and, at the same time, great penetration and ingenuity; he is quite an original thinker, and his mind is evidently stamped with the signet of true philosophy.

The success with which he has examined some of the most obscure phenomena of life, pointing out their association and harmony, and their connexion with some general laws, which seem to pervade and influence the vitality of all animals, from the lowest to the highest—the admirable fitness of his experiments, the ingenuity of their contrivance, and the skill of their execution—the strict and logical accuracy of most of his inductions, and the practical importance of many, fully warrant our high encomiums. Moreover, in addition to his own immediate discoveries, he has thrown much light on the researches of others, and has been enabled to reconcile most happily, the conflicting and discrepant views of different authors. There is so much sound truth and acute observation in the following passage, that we cannot refrain from directing the reader's attention to it.

"From our being accustomed to find a constancy in the phænomena of inorganic nature, and habituated to judge of the truth of the results of experiment by the possibility of our reproducing them at pleasure, we are led to seek with anxiety for the same character, in an order of facts which are necessarily variable. Hence the difficulty of obtaining general assent to the results of physiological experiments, which by their nature are precluded from offering that uniformity on which the mind reposes with confidence.

Convinced that different and even opposite results do not necessarily exclude each other, when vitality is concerned, I have always endeavoured so to vary my experiments, that I might reproduce some of the phænomena which appear contradictory in the works of other physiologists." 226.

To Dr. Hodgkin great praise is due, for the manner in which he has executed his task; but he might have rendered the work more saleable, and, therefore, more generally useful, had he shorn it of some extraneous matter. To introduce an inaugural thesis, and a juvenile essay, savours rather too much of what, in parliamentary language, is termed "adding a rider to a money bill," by which the Commons, as is well known, frequently manage to obtain the sanction of the Lords and King to a measure, by affixing it to any grant of supplies; and we regret it the more, because it has prevented him, we are told, from publishing the copious tables of Dr. Edwards, appended to each section, and in which the individual results of the very numerous experiments, and the conclusions to be drawn from them, were specially detailed. Surely the acquisition of these would have been more valuable than a Latin thesis on Absorption, various essays, on Atmospheric

Electricity, the Composition of Animal Fluids, the Uses of the Spleen, and so forth.

In the event of a second edition, or of an abridgement, we should suggest to Dr. Hodgkin several alterations. Instead of merely translating the original memoirs of Dr. Edwards, which were, as a matter of course, disjointed, and frequently unconnected, they should have been all brought together and compared with each other, selecting whatever was novel and important, and rejecting whatever was superfluous, and had been already explained; much repetition would have been thus avoided, and much misplaced matter would have been made more available. For example, had the subject of respiration been treated of by itself, and its modifications traced in the various classes of animals, from the batrachia to the perfect mammalia, thus exhibiting how many differences there are in different animals, but yet how these very differences harmonize, and are referable to a simple and uniform law of function, we should have had a most interesting and instructive discourse, instead of being quickly led from one topic to another, whereby the mind is bewildered and confused; in short, had the various chapters been headed thus—"Respiration," "Production of Heat," "Asphyxia," and so forth, in place of their present titles—"Batrachian Animals," "Fishes and Reptiles," "Warm-blooded Animals," &c. &c. each would have been valuable separately, and in connexion with the others. As we have already said, we are aware that this arrangement arose from each individual chapter having been originally a distinct memoir, read to the Academy of Sciences; but the translator would have acted wisely, had he become an analyser and digester at the same time. It appears quite unnecessary to introduce, upon all occasions, reference both to Fahrenheit's and to the centigrade thermometer, as any reader can at once convert the denominations of one into those of the other: considerable confusion has in consequence crept in, as when the term zero, at page 17, is used, the reader may very easily fall into the mistake of supposing that it alludes to 32 degrees below the point of freezing. The very opposite error is committed, in regard to the measurement, at page 292, for the French terms only are given, and no reference to the corresponding English ones. In many parts, the language is rather obscure and ungraceful, so that sentences here and there are nearly unintelligible. On the whole, however, the medical public is greatly indebted to Dr. Hodgkin for the present work; and, though susceptible of improvement, it ought to be well studied by every philosophical physician and surgeon.

